# Investigating the causal impact of polycystic ovary syndrome on gestational diabetes mellitus: a two-sample Mendelian randomization study

**DOI:** 10.3389/fendo.2024.1337562

**Published:** 2024-02-05

**Authors:** Guan Guixue, Pu Yifu, Tang Xiaofeng, Sun Qian, Gao Yuan, Yang Wen, Han Conghui, Zhu Zuobin

**Affiliations:** ^1^ Department of Gynecology, The First People’s Hospital of Lianyungang, Lianyungang, Jiangsu, China; ^2^ Department of Gynecology, The Affiliated Lianyungang Hospital of Xuzhou Medical University, Lianyungang, Jiangsu, China; ^3^ Department of Gynecology, The First Affiliated Hospital of Kangda College of Nanjing Medical University, Lianyungang, Jiangsu, China; ^4^ Laboratory of Genetic Disease and Perinatal Medicine, Key Laboratory of Birth Defects and Related Diseases of Women and Children, Ministry of Education, West China Second University Hospital, Sichuan University, Chengdu, Sichuan, China; ^5^ Prenatal Diagnosis Center, West China Second University Hospital, Sichuan University, Chengdu, Sichuan, China; ^6^ Department of Urology, Xuzhou Central Hospital, Xuzhou, Jiangsu, China; ^7^ Department of Urology, Xuzhou Clinical School of Xuzhou Medical University, Xuzhou, Jiangsu, China; ^8^ Xuzhou Engineering Research Center of Medical Genetics and Transformation, Key Laboratory of Genetic Foundation and Clinical Application, Department of Genetics, Xuzhou Medical University, Xuzhou, Jiangsu, China

**Keywords:** polycystic ovary syndrome, gestational diabetes mellitus, causal effects, Mendelian randomization study, genome-wide association studies

## Abstract

**Introduction:**

Determining the causal relationship between polycystic ovary syndrome (PCOS) and gestational diabetes mellitus (GDM) holds significant implications for GDM prevention and treatment. Despite numerous observational studies suggesting an association between PCOS and GDM, it remains unclear whether a definitive causal relationship exists between these two conditions and which specific features of PCOS contribute to increased incidence of GDM.

**Methods:**

The causal relationship between polycystic ovary syndrome (PCOS), its characteristic indices, and gestational diabetes mellitus (GDM) was investigated using a two-sample Mendelian randomization study based on publicly available statistics from genome-wide association studies (GWAS). The inverse-variance weighted method was employed as the primary analytical approach to examine the association between PCOS, its characteristic indices, and GDM. MR Egger intercept was used to assess pleiotropy, while Q values and their corresponding P values were utilized to evaluate heterogeneity. It is important to note that this study adopts a two-sample MR design where PCOS and its characteristic indices are considered as exposures, while GDM is treated as an outcome.

**Results:**

The study results indicate that there is no causal relationship between PCOS and GDM (all methods P > 0.05, 95% CI of OR values passed 1). The IVW OR value was 1.007 with a 95% CI of 0.906 to 1.119 and a P value of 0.904. Moreover, the MR Egger Q value was 8.141 with a P value of 0.701, while the IVW Q value was also 8.141 with a P value of 0.774, indicating no significant heterogeneity. Additionally, the MR Egger intercept was 0.0004, which was close to zero with a P value of 0.988, suggesting no pleiotropy. However, the study did find a causal relationship between several other factors such as testosterone, high-density lipoprotein, sex hormone-binding globulin, body mass index, waist-hip ratio, apolipoprotein A-I, number of children, diabetes illnesses of mother, father and siblings, hemoglobin A1c, fasting insulin, fasting blood glucose, years of schooling, and GDM based on the IVW method.

**Conclusion:**

We observed no association between genetically predicted PCOS and the risk of GDM, implying that PCOS itself does not confer an increased susceptibility to GDM. The presence of other PCOS-related factors such as testosterone, high-density lipoprotein, and sex hormone-binding globulin may elucidate the link between PCOS and GDM. Based on these findings, efforts aimed at preventing GDM in individuals with PCOS should prioritize those exhibiting high-risk features rather than encompassing all women with PCOS.

## 1 Introduction

Polycystic Ovary Syndrome (PCOS) is a female endocrine disorder characterized by unclear etiology and highly heterogeneous clinical manifestations. It is marked by infrequent ovulation or anovulation, clinical and/or biochemical hyperandrogenism, and polycystic ovaries with cyst-like changes in the ovaries (1). The prevalence of PCOS ranges from approximately 5% to 20% (2). The Rotterdam criteria were established as the most commonly employed diagnostic guidelines for identifying PCOS (3). According to this standard, a diagnosis of PCOS is typically made if an individual meets at least two out of the following three criteria: 1) irregular or absent ovulation (Oligoovulation or Anovulation, O); 2) the presence of clinical and/or biochemical indicators of elevated androgen levels (Hyperandrogenemia, H), and 3) the identification of polycystic ovarian morphology through ultrasound (Polycystic Ovaries, P). PCOS is categorized into four types: Type A (H+O+P), Type B (O+H), type C (H+P) and type D (O+P). The distribution of each PCOS subtype varies across different racial and study populations. Individuals with PCOS commonly experience metabolic abnormalities, such as irregularities in glucose tolerance and disorders in lipid metabolism. Following pregnancy, PCOS patients exhibit a notable increase in perinatal complications. The extent of these complications varies based on factors like different phenotypes, ethnicities, personal and family histories, psychological well-being, lifestyle, and other individual characteristics, resulting in diverse manifestations of reproductive abnormalities.

Gestational Diabetes Mellitus (GDM) refers to elevated blood sugar levels occurring during pregnancy. Typically, insulin needs increase during pregnancy to meet the body’s demand for glucose. For some women, this additional demand can lead to higher-than-normal blood sugar levels, known as GDM. This condition often manifests in the later stages of pregnancy and usually resolves to normal levels after delivery (4, 5). The definition of GDM has undergone recent changes. Like one commentary, there’s a call for establishing new diagnostic criteria for GDM (6). GDM is the prevailing metabolic disorder, potentially impacting as many as 25% of women during pregnancy (7, 8). There are reports indicating a frequent association between PCOS and a heightened risk of developing GDM (9–17). Women with PCOS who smoke are at a higher risk of developing GDM (18). Besides, a meta-analysis of 29 observational studies that compared pregnancy outcomes in women undergoing *in vitro* fertilization (IVF) indicated that women with PCOS were more likely to experience GDM, with an odds ratio (OR) value of 2.67 (19). According to another meta-analysis, women with PCOS are more likely to develop GDM compared to those without PCOS (20). The occurrence of GDM in different types of PCOS patients varied, with the highest incidence observed in Type A (54.7%), followed by Type C (32.6%), Type D (7.4%), and Type B (5.3%) (21).

While PCOS is recognized as an independent risk factor for GDM, it’s important to note that not all women with PCOS necessarily develop GDM (22). The elevated risk of GDM in individuals with PCOS is not solely dependent on obesity. However, obesity and the related insulin resistance (IR) can exacerbate this risk (23, 24). In contrast to individuals with a normal weight and PCOS, those with obesity and PCOS exhibit a higher prevalence of GDM. Additionally, they tend to experience more pronounced IR, elevated fasting insulin levels, and increased IR and secretion indices before pregnancy (25). In comparison to the normal control group, pregnant women with PCOS are more prone to excessive weight gain during both early pregnancy and throughout the entire pregnancy. This heightened weight gain contributes to an increased risk of GDM (26). Findings from a community-based longitudinal study tracking the body mass index (BMI) trajectory in women of reproductive age indicate that both BMI and PCOS are linked to an elevated prevalence of GDM. Moreover, the risk of GDM is notably higher when both factors coexist (27).

Furthermore, the study highlighted a strong association between GDM and factors such as overweight, obesity, a history of GDM in the mother, a family history of Type 2 diabetes mellitus (T2DM) in both the subject’s parents, and a history of preterm birth (28). A predictive model has been formulated to proactively prevent GDM in women diagnosed with PCOS (29). A study investigating predictors of GDM in Chinese PCOS patients identified pre-pregnancy weight gain, pre-pregnancy waist-to-hip ratio, IR index, and sex hormone-binding globulin level at 24 weeks of gestation as independent risk factors for GDM (30). Elevated levels of β2-microglobulin (β2-MG) and cystatin C, along with a high albumin-to-creatinine ratio (ACR), could serve as risk factors for Chinese women with PCOS and GDM during mid-pregnancy, as indicated by a study (31). For individuals with PCOS, the continuous use of metformin throughout pregnancy is linked to a substantial ninefold reduction (from 30% to 3.44%) in the incidence of GDM (32). Another study has demonstrated a robust connection between various early-pregnancy risk factors and the development of GDM in women with PCOS. These risk factors primarily involve the regulation of glucose, lipid, and androgen metabolism. Notably, factors such as fasting plasma glucose (FPG), non-high-density lipoprotein-cholesterol, and sex hormone-binding globulin are predictive of the onset of GDM (33).

Despite the numerous similarities between PCOS and GDM, such as insulin resistance and their link to type 2 diabetes and postpartum impaired glucose metabolism (34), their relationship has mainly been observed through observational studies. Nevertheless, it is still uncertain whether there exists a causal link between PCOS and GDM, and which specific characteristics of PCOS contribute to the heightened incidence of GDM. To address these uncertainties, Mendelian randomization (MR) offers a valuable approach to deduce causality and offer reliable estimates of the connection between exposure and outcome. Unlike observational studies, MR minimizes the impact of confounding factors and reverse causation by utilizing single nucleotide polymorphisms (SNPs) identified from Genome-Wide Association Studies (GWAS) as instrumental variables (IVs). Consequently, this study is designed to explore the causal relationship between PCOS, its diverse features, and GDM through MR analysis.

## 2 Methods

### 2.1 Study design

**Figure 1:**
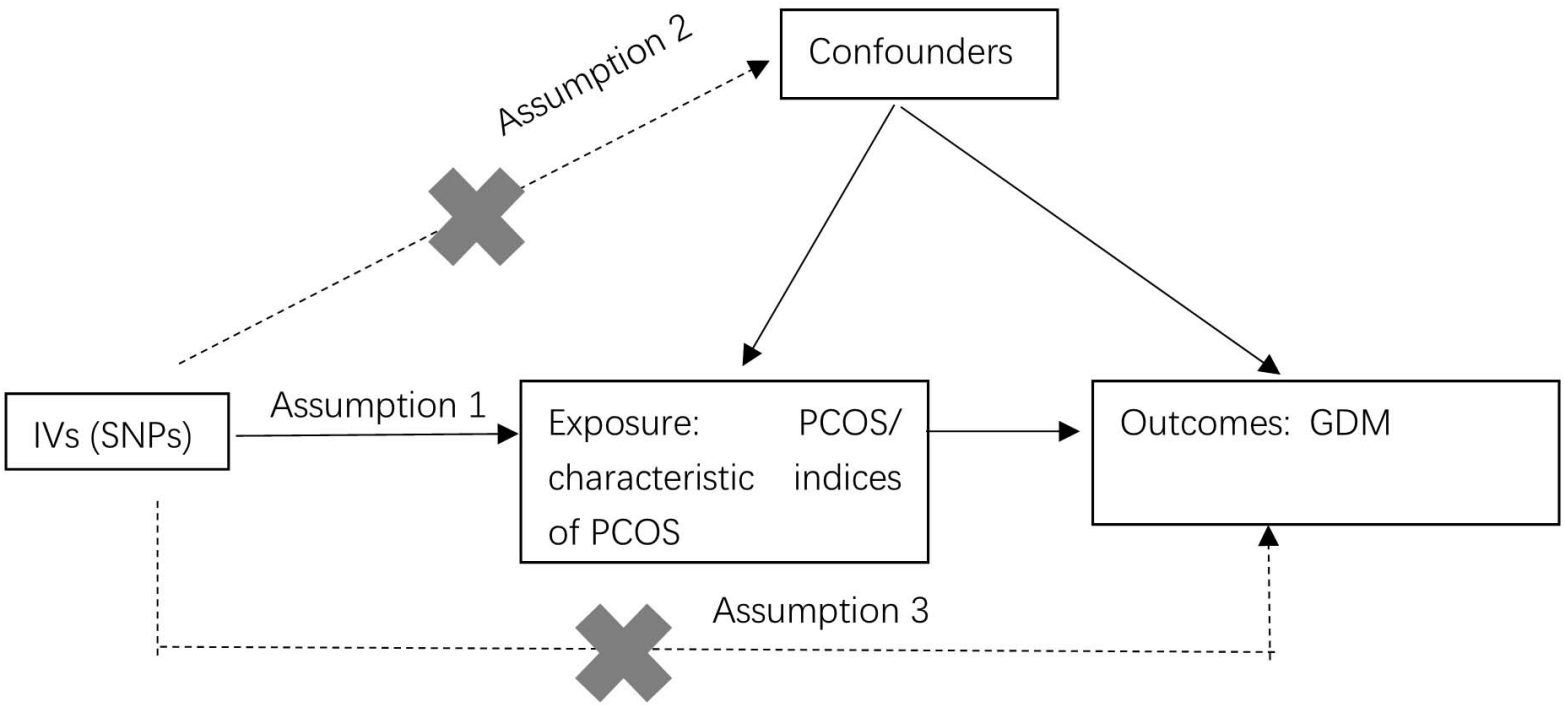
Flow chart of the Two-sample MR study design.

The study employed a two-sample MR design to investigate the causal relationships between PCOS, the characteristic indices of PCOS, and GDM (as depicted in **Figure 1**). The MR analysis was based on three key hypotheses: 1) SNPs identified in GWAS served as IVs. These SNPs were chosen because they exhibited a strong correlation with the exposure. 2) The selected IVs were ensured not to be associated with any confounding factors that could potentially skew the results. 3) The IVs were assumed to impact the outcomes (GDM) solely through their association with the exposure (PCOS or the characteristic indices of PCOS). In other words, the study aimed to establish a direct and causal link between PCOS, the characteristic indices of PCOS and GDM.

### 2.2 Data sources and selection of IVs

The PCOS GWAS originates from a meta-analysis that amalgamates data from seven European cohort studies, including a subset of self-reported PCOS cases. This presents challenges in objectively evaluating factors like medication intake, specific medications used, and related variables such as pregestational obesity among the included PCOS patients before analysis, potentially introducing bias. In contrast, other studies incorporated into the GWAS rigorously screen patients under researcher supervision, ensuring robust safeguards. Furthermore, this meta-analysis of PCOS GWAS boasts a considerable and representative sample size. The GWAS on PCOS comprised 10,074 cases and 103,164 controls (35). The source cohorts of the PCOS GWAS and their characteristics, especially the definition of PCOS, the number and percentage of individuals with clinical or biochemical hyperandrogenism, ovulatory dysfunction, and polycystic ovarian morphology, are displayed in **Table 1**. Besides, it’s crucial to highlight that the understanding of GDM has evolved in recent years. Several instances identified as GDM actually pertain to women who had type 2 diabetes mellitus before becoming pregnant. The criteria used to screen for GDM differ worldwide and can vary even within a specific country. The authoritative organizations for GDM include the World Health Organization (WHO), the International Association of Diabetes and Pregnancy Study Groups (IADPSG), and the International Federation of Gynecology and Obstetrics (FIGO). For GDM, the GWAS used in this study involved 5,687 GDM cases and 117,892 controls sourced from the IEU Open GWAS database (ID: finn-b-GEST_DIABETES), which has been widely utilized in different Mendelian randomization studies. The GDM GWAS was conducted based on the WHO criteria (75g Oral Glucose Tolerance Test: fasting blood glucose greater than or equal to 5.1 mmol/L, 1-hour post-ingestion greater than or equal to 10.0 mmol/L, or 2-hour post-ingestion greater than or equal to 8.5 mmol/L. High blood glucose at one or more time points above the criteria confirms the diagnosis). All individuals in both studies were of European descent. Fourteen independent SNPs of PCOS were used according to a previous article (36). The criterions of selection of IVs were as follows: independent SNPs (r^2^ < 0.001 and clumping distance > 10,000 kb); P value < 5 × 10^− 8^. The F statistics for all SNPs incorporated into the MR analysis were assessed using mRnd, an online tool available at https://shiny.cnsgenomics.com/mRnd/. Importantly, all F statistics for the included SNPs exceeded the threshold of 10.

**Table 1:** The source cohorts of the PCOS GWAS and their characteristics.

Cohort	PCOS definition	Clinical or biochemical hyperandrogenism n (%)	Ovulatory dysfunction n (%)	Polycystic ovarian morphology n (%)
Rotterdam	NIH and Rotterdam	439 (37.0)	946 (79.8)	661 (55.8)
UK (London/ Oxford)	NIH and Rotterdam	455 (67.9)	537 (80.1)	383 (57.2)
EGCUT	Rotterdam	NA	NA	NA
deCODE	NIH and Rotterdam	644 (97.9)	380 (57.7)	507 (77.1)
Chicago	NIH	984 (100)	984 (100)	NA
Boston	NIH	485 (100)	485 (100)	441 (90.9)
23andMe	Self report (defined by questionnaire)	NA	NA	NA

Results are reported as a number (%).

NA, not available; NIH, The National Institutes of Health; PCOS, polycystic ovary syndrome; GWAS, Genome-Wide Association Study.

### 2.3 MR analysis

In this investigation, the primary analytical approach employed was the inverse-variance weighted (IVW) method, focusing on assessing the connection between PCOS, the characteristic indices of PCOS, and GDM. The study utilized the MR Egger intercept to evaluate pleiotropy. Heterogeneity was assessed using Q values and their associated P values. It’s crucial to highlight that this is a two-sample MR study, treating PCOS and the characteristic indices of PCOS as the exposure and GDM as the outcome. Funnel plots were created to identify potential outlier SNPs. The causal effects of PCOS and the characteristic indices of PCOS on GDM were expressed using odds ratios (ORs) and 95% confidence intervals (CIs). The R software (version 4.2.1) two-sample MR package was utilized for all analyses. Statistically significant causality was considered when the P-value was less than 0.05.

## 3 Results

### 3.1 Causal association between PCOS and GDM according to five methods

**Figure 2:**
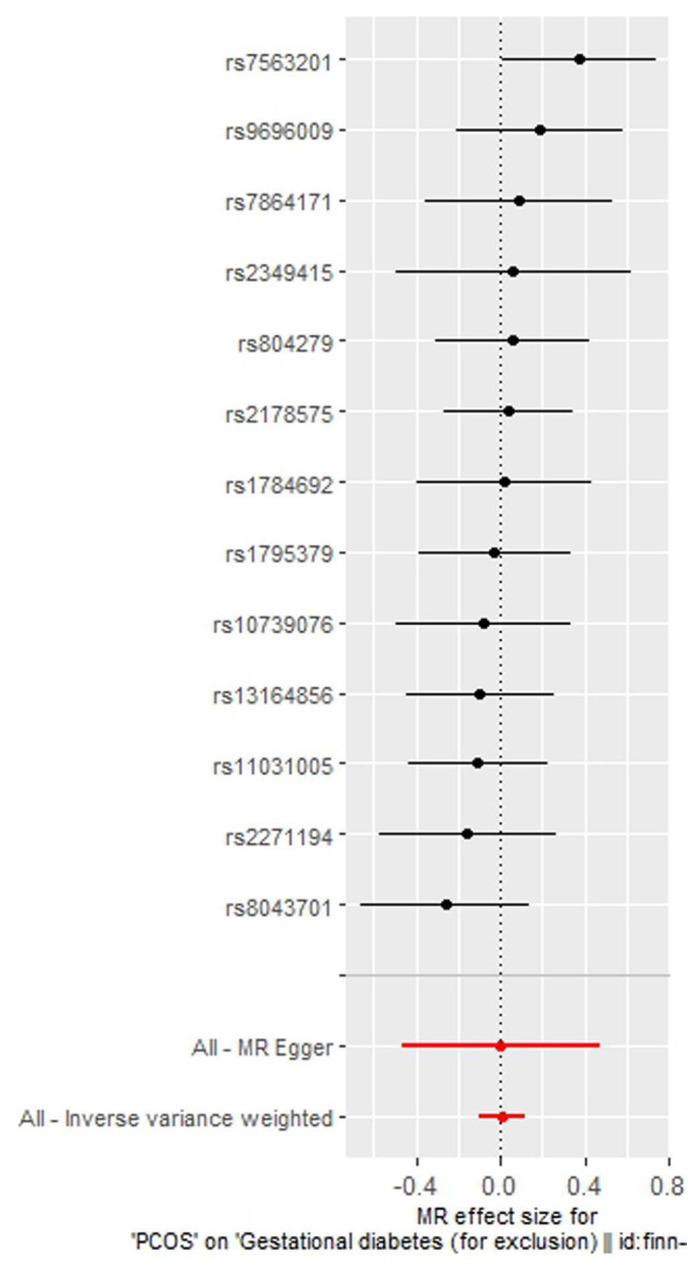
MR effect size for polycystic ovary syndrome on gestational diabetes mellitus.

**Figure 3:**
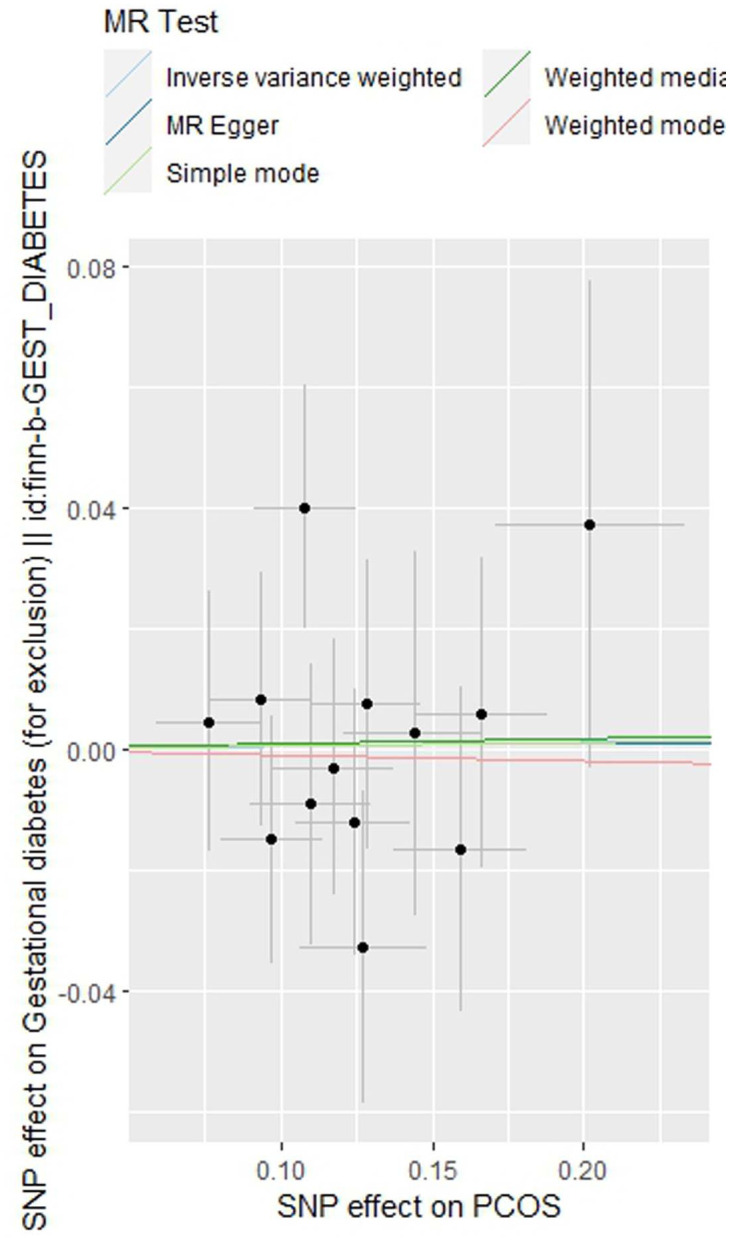
Scatter plot of the MR analysis of polycystic ovary syndrome on gestational diabetes mellitus.

**Figure 4:**
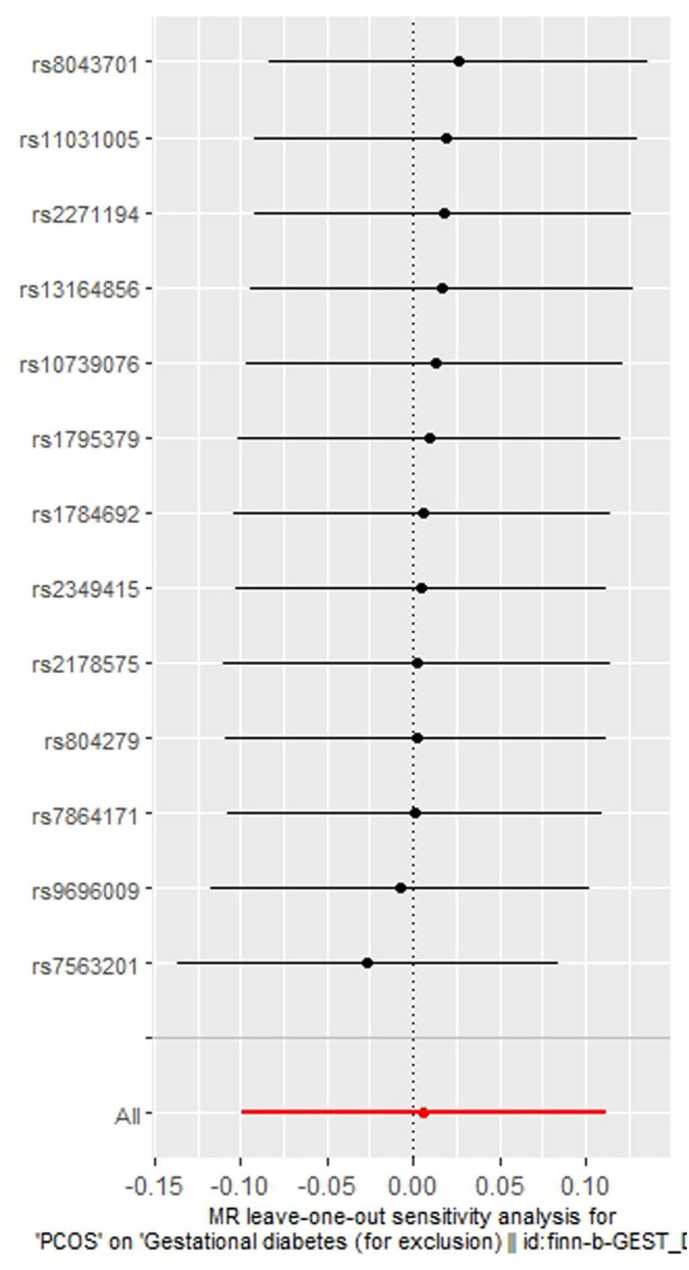
Leave-one-out regression analysis of polycystic ovary syndrome on gestational diabetes mellitus.

**Figure 5:**
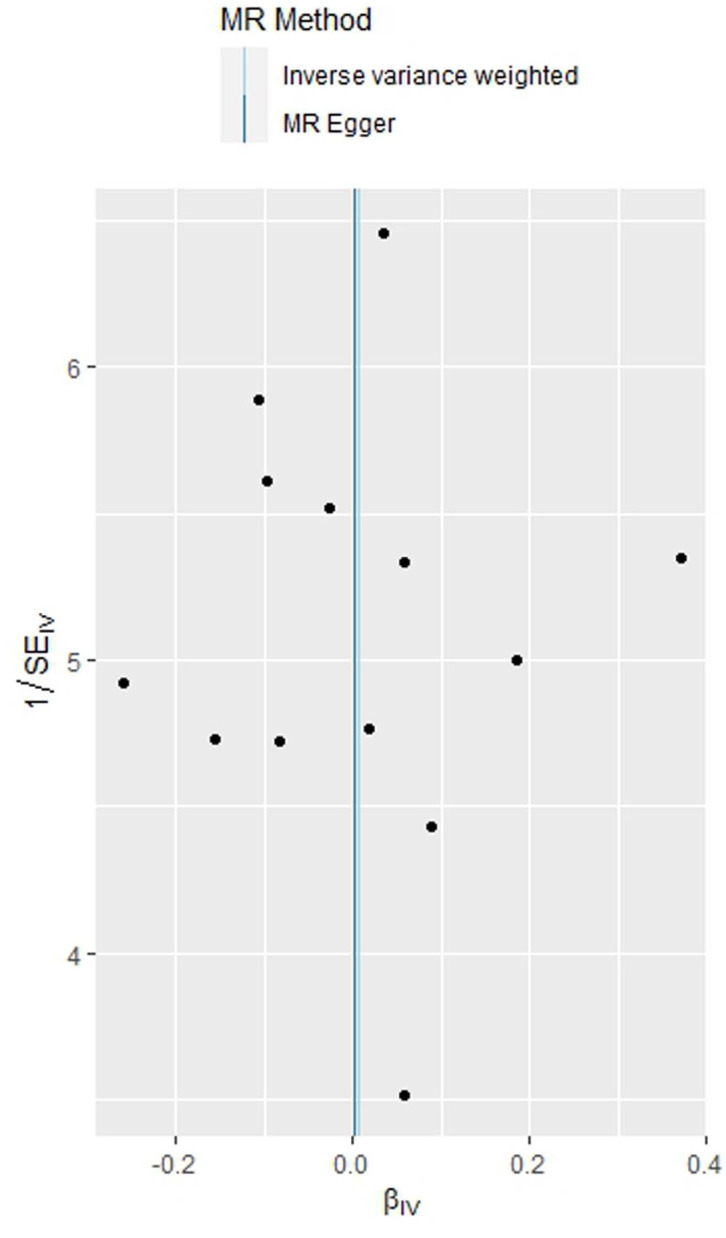
Funnel plot of the MR analysis of polycystic ovary syndrome on gestational diabetes mellitus.

As indicated in **Table 2**, the analysis using the five methods did not reveal a causal relationship between PCOS and GDM. The causal effect between PCOS and GDM was not supported (IVW OR:1.007, 95%CI: 0.906 ~ 1.119, P=0.904). For heterogeneity, MR egger Q value was 8.141, P= 0.701; IVW Q value was 8.141, P= 0.774. For pleiotropy, MR egger intercept was 0.0004, near 0, P =0.988. **Figures 2**–**5** present the MR effect sizes for PCOS on GDM, along with scatter plots, leave-one-out plots, and funnel plots, respectively. Sensitivity analysis of leave-one-out showed no significant outlier SNP. Funnel plot was roughly symmetrical without obvious heterogeneity. No Pleiotropy was found. Results are robust and the conclusion is reliable.

**Table 2:** Causal association between PCOS and GDM (ieu ID: finn-b-GEST_DIABETES).

Methods	IVs (n SNPs)	Beta	SE	P	OR	95%CI
MR Egger	13	0.003	0.237	0.991	1.003	0.630, 1.595
Weighted median	13	0.009	0.073	0.904	1.009	0.875, 1.164
Inverse variance weighted	13	0.007	0.054	0.904	1.007	0.906, 1.119
Simple mode	13	0.005	0.118	0.965	1.005	0.797, 1.268
Weighted mode	13	-0.010	0.117	0.934	0.990	0.787, 1.246

PCOS, polycystic ovary syndrome; GDM, gestational diabetes mellitus; SNP, single nucleotide polymorphisms; IVs, instrumental variables; OR, odds ratio; CI, confidence interval; SE, standard error; n, number.

### 3.2 Causal association between various characteristics indices of PCOS and GDM: based on IVW method

As shown in **Table 3**, the characteristic indices of PCOS showed a causal relationship with GDM (based on different IVs, OR values, and 95% CI; all P values were less than 0.05). There was a causal relationship between testosterone, high-density lipoprotein, sex hormone-binding globulin, body mass index, waist-hip ratio, apolipoprotein A-I, number of children, illnesses of mother, father and siblings: diabetes, hemoglobin A1c, fasting insulin, fasting blood glucose, years of schooling and GDM according to the IVW method.

**Table 3:** The associations between genetically predicted characteristics indices of PCOS and the risk of GDM.

Exposure	GWAS ID	Outcome	n SNPs	Method	OR (95%CI)	P value
T	ebi-a-GCST90012104	GDM	96	IVW	1.364(1.033 – 1.800)	0.028
HDL	ieu-b-109	GDM	315	IVW	0.752(0.675 – 0.837)	2.038e-7
SHBG	ieu-b-4870	GDM	176	IVW	0.789(0.686 – 0.908)	0.001
BMI	ukb-b-19953	GDM	418	IVW	1.730(1.515 – 1.976)	6.448e-9
Waist-hip ratio	ieu-b-4830	GDM	13	IVW	1.785e+5(749.753 – 4.249e+7)	1.485e-5
Apo A-1	ieu-b-107	GDM	261	IVW	0.768(0.681 – 0.865)	1.487e-5
Number of children	ieu-b-4760	GDM	7	IVW	6.648(1.372 – 32.227)	0.019
Illnesses of mother: Diabetes	ukb-b-16451	GDM	22	IVW	13331.85(677.689 - 262271)	4.142e-10
Illnesses of father: Diabetes	ukb-b-20211	GDM	26	IVW	32662.140(2163.912 – 493003.2)	6.119e-14
Illnesses of siblings: Diabetes	ukb-b-18042	GDM	15	IVW	1.109e+5(3.862e+3 - 3184907)	1.191e-11
HbA1c	ieu-b-4842	GDM	24	IVW	1.352(1.003 – 1.821)	0.048
Fasting insulin	ebi-a-GCST90002238	GDM	38	IVW	2.965(1.143 – 7.696)	2.549e-2
Fasting blood glucose	ebi-a-GCST008032	GDM	7	IVW	3.199(1.371 – 7.462)	0.007
Years of schooling	ieu-a-1239	GDM	295	IVW	0.657(0.534 – 0.808)	6.887e-5

PCOS, polycystic ovary syndrome; T, testosterone; HDL, high-density lipoprotein; SHBG, sex hormone-binding globulin; BMI, body mass index; Apo A-1, apolipoprotein A-I; HbA1c, Hemoglobin A1c; SNP, single nucleotide polymorphism; GWAS, genome-wide association studies; IVW, inverse variance weighted; n, number.

## 4 Discussion

It’s crucial to highlight that despite the numerous similarities between PCOS and GDM, such as insulin resistance and their association with type 2 diabetes and postpartum impaired glucose metabolism (34), their relationship has been observed primarily through observational studies, lacking conclusive evidence of a causal link. Presently, there are numerous MR studies on PCOS (37–41). To the best of our knowledge, this is the initial study investigating the causal effects of PCOS and its characteristic indices on GDM. In this investigation, phenotypic GWAS data were scrutinized using two-sample MR, revealing no conclusive evidence of a causal relationship between PCOS and GDM, which is as expected due to the numerous similarities between two conditions. However, a causal association was identified between several other factors, including testosterone, high-density lipoprotein, sex hormone-binding globulin, body mass index, waist-hip ratio, apolipoprotein A-I, number of children, illnesses of parents and siblings (diabetes), hemoglobin A1c, fasting insulin, fasting blood glucose, years of schooling, and GDM. The PCOS GWAS data, derived from a comprehensive meta-analysis, involved 10,074 cases and 103,164 controls of European ancestry diagnosed with PCOS based on NIH criteria (2,540 cases and 15,020 controls), Rotterdam criteria (2,669 cases and 17,035 controls), or self-reported diagnosis (5,184 cases and 82,759 controls). Within this meta-analysis, **Table 1** outlines the definitions of PCOS patients, the case-control distribution, and the proportions of PCOS cases exhibiting hyperandrogenism, ovulatory dysfunction, and polycystic ovarian morphology. It’s crucial to note that some cases were self-reported, potentially affecting the data extraction process. As we all know, obesity and/or PCOS are high-risk factors in the development of GDM (42). Whether pregestational obesity and any pharmacological therapy were an exclusion criterion for all PCOS patients, there could be a confounding factor.

The amalgamation of data from various study cohorts in this meta-analysis currently impedes the categorization of PCOS based on subtypes. As of now, it’s feasible to present data within each cohort, highlighting the presence of hyperandrogenism, ovulatory dysfunction, and polycystic ovarian morphology. Recognizing that certain PCOS phenotypes (A and B) often manifest more severe metabolic traits compared to others (C and D), the latter phenotypes typically exhibit mild or minimal metabolic irregularities (2). This distinction suggests that diabetes or cardiovascular disease screening might be unnecessary for C and D phenotypes. Therefore, establishing categories such as A, B, C, and D subtypes within PCOS would be incredibly valuable, particularly for focusing on individuals with A and B subtype PCOS, holding significant clinical implications. Looking forward, there is anticipation for future GWAS studies focused on different PCOS subtypes.

In our research, the GWAS exploring GDM integrated data obtained from the IEU Open GWAS database, identified by the ID finn-b-GEST_DIABETES, involving 5,687 individuals diagnosed with GDM and 117,892 controls. This specific GWAS has been widely utilized in different Mendelian randomization studies, notably referenced in the recent publication article (43). Nevertheless, it’s important to note that the definition of GDM has changed over the past few years. Many cases reported as GDM involve women who had type 2 diabetes mellitus before pregnancy. The screening criteria for GDM differ globally and may even vary within a single country. Therefore, similar to PCOS GWAS, we aim for future GDM GWAS studies with more rigorous inclusion and exclusion criteria, as well as larger sample sizes.

Women diagnosed with PCOS commonly exhibit inherent IR, a condition observed in as many as 80% of individuals with PCOS (24, 44). Despite the metabolic disruptions linked to PCOS, establishing PCOS as an autonomous risk factor for GDM continues to be challenging (45, 46). The initial meta-analysis conducted in 2006, encompassing 720 women with PCOS, revealed that individuals with PCOS faced an almost threefold increase in the likelihood of developing GDM (45). Subsequent meta-analyses conducted in 2011 (47) and 2013 (46) provided further confirmation of the elevated risks associated with PCOS women in developing GDM. Nevertheless, both of these analyses noted substantial heterogeneity within their findings, primarily attributed to variations in study designs and diverse ethnic backgrounds. Additionally, numerous included studies lacked the statistical power to adequately control for potential confounding effects, with obesity being the most noteworthy among them (46, 47). In 2016, Yu et al. conducted an extensive meta-analysis involving over 17,000 women with PCOS. The findings of this study conclusively asserted that PCOS stands as a primary risk factor for adverse pregnancy outcomes (48). Subsequent meta-analyses have emerged to investigate pregnancy outcomes in women with PCOS. Since most studies on the risk of GDM in PCOS patients are retrospective, there is inconclusive evidence regarding whether PCOS itself is a direct risk factor for GDM or if other associated factors play a role (7, 20, 24, 49). Certainly, the incomplete comprehension of the interplay between PCOS and the onset of GDM can be attributed to the intricate and multifaceted origins of PCOS. The diverse array of potential factors influencing pregnancy complications, coupled with the varied methodologies employed in conducted studies, likely contributes to these significant knowledge gaps.

PCOS escalates the likelihood of various pregnancy complications, with GDM being one of them. Expectant mothers with PCOS and simultaneous IR are at an increased risk of developing GDM (50). Nevertheless, research indicates that IR tends to be more prevalent in overweight women with PCOS. Moreover, obesity itself is recognized as an independent risk factor for GDM during pregnancy (28). It’s not unexpected that ongoing studies examining the risk of GDM in pregnant women with PCOS face challenges in effectively controlling for obesity within this population. Consequently, the existing literature is still in contention regarding whether PCOS independently poses a risk for GDM development or if it is, in fact, the underlying (and associated) obesity that contributes to the additional risk (28).

This study utilized 14 characteristic indices of PCOS and large-sample PCOS GWAS data from individuals of the same ethnic background—European ancestry. The proposed method carries several advantages. Firstly, PCOS itself does not inherently increase the risk of GDM; therefore, characteristic indices of PCOS were employed to investigate their causal effects on GDM. Some indices related to PCOS characteristics were found to have causal effects on GDM. PCOS is a diverse endocrine disorder, encompassing symptoms such as hyperandrogenemia, glucose and lipid metabolism disorders, obesity, waist-to-hip ratio imbalance, menstrual irregularities, ovulation abnormalities, and more. Secondly, this study furnishes evidence supporting the importance of regulating glycemic and lipid metabolism, controlling body weight, and reducing hyperandrogenemia in individuals with PCOS, aiming to mitigate the occurrence of GDM. Despite the noteworthy results uncovered in this study, it is important to acknowledge its limitations. Firstly, the GWAS data were derived from individuals of European ancestry, and the generalizability of these findings to other ethnicities requires further investigation. Additionally, some analyses involved a small number of SNPs, fewer than 10, potentially leading to less precise results and compromising confidence. Continuous updates and releases of GWAS data on PCOS may help address these limitations in the future. Finally, the accuracy of the conclusions might be enhanced if measures of characteristics indices of PCOS were restricted to female participants only.

## 5 Conclusion

To summarize, this two-sample MR study suggests that genetically predicted PCOS is not significantly associated with GDM. PCOS itself does not independently contribute to an increased risk of GDM; instead, the elevated GDM risk in PCOS is linked to other potential factors, including testosterone, high-density lipoprotein, sex hormone-binding globulin, body mass index, waist-hip ratio, apolipoprotein A-I, number of children, illnesses of parents and siblings (diabetes), hemoglobin A1c, fasting insulin, fasting blood glucose, years of schooling. It is essential to focus on regulating glycemic and lipid metabolism, controlling body weight, and reducing hyperandrogenemia to decrease the occurrence of GDM in individuals with PCOS. Further scientific studies are required to unveil the mechanisms underlying the heightened risk of GDM in PCOS patients.

## Data availability statement

The original contributions presented in the study are included in the article. Further inquiries can bedirected to the corresponding author.

## Ethics statement

Ethical approval was not required for the study involving humans in accordance with the local legislation and institutional requirements. Written informed consent to participate in this study was not required from the participants or the participants’ legal guardians/next of kin in accordance with the national legislation and the institutional requirements.

## Author contributions

GG: Conceptualization, Formal analysis, Writing – original draft, Writing – review & editing. PY: Conceptualization, Formal analysis, Methodology, Software, Validation, Writing – original draft, Writing – review & editing. TX: Investigation, Software, Writing – review & editing. SQ: Data curation, Investigation, Methodology, Writing – review & editing. GY: Data curation, Investigation, Methodology, Writing – review & editing. YW: Funding acquisition, Validation, Writing – review & editing. HC: Writing – review & editing. ZZ: Funding acquisition, Supervision, Writing – review & editing.
